# Analysis of the relationship between prescribed dose and dosimetric advantage of real-time intraoperatively built custom-linked seeds in iodine-125 prostate brachytherapy

**DOI:** 10.1186/s13014-017-0932-7

**Published:** 2017-12-01

**Authors:** Katsumi Hirose, Masahiko Aoki, Mariko Sato, Hiroyoshi Akimoto, Yasuhiro Hashimoto, Atsushi Imai, Noritaka Kamimura, Hideo Kawaguchi, Yoshiomi Hatayama, Ichitaro Fujioka, Mitsuki Tanaka, Chikara Ohyama, Yoshihiro Takai

**Affiliations:** 1 Department of Radiology and Radiation Oncology, Hirosaki University Graduate School of Medicine, 5 Zaifu-cho, Hirosaki, Aomori, 036-8562 Japan; 2 Department of Urology, Hirosaki University Graduate School of Medicine, 5 Zaifu-cho, Hirosaki, Aomori, 036-8562 Japan; 3 Department of Radiation Oncology, Southern Tohoku BNCT Research Center, 7-10, Yatsuyamada, Koriyama, Fukushima, 963-8052 Japan

**Keywords:** Prostate cancer, Brachytherapy, IBCL seed, Seed migration

## Abstract

**Background:**

The purpose of this study was to investigate the differences in the dosimetric advantage of using intraoperatively built custom-linked (IBCL) seeds between permanent iodine-125 (I-125) seed implantation (PI) alone and PI followed by external-beam radiation therapy (EBRT) for prostate cancer.

**Methods:**

We reviewed the records of 62 patients with localized prostate cancer who received transperineal interstitial brachytherapy with I-125 using free seeds or IBCL seeds. Twenty-four low- and intermediate-risk patients underwent PI alone with the prescribed dose of 160 Gy, and 39 high-risk patients underwent PI with 110 Gy, followed by EBRT with 45 Gy (PI + EBRT). Intraoperative and post-implant dosimetric parameters 1 month after implantation were collected and analyzed.

**Results:**

The numbers of patients implanted with free seeds and IBCL seeds were 14 (58.3%) and 10 (41.7%), respectively, in the PI group and 25 (65.8%) and 13 (34.2%), respectively, in the PI + EBRT group. In the PI group, although there were significant differences in prostate V100 (*p* = 0.003) and D90 (*p* = 0.009) and rectum V100 (*p* = 0.026) on intraoperative dosimetry, these differences were not found on post-implant dosimetry. In the PI + EBRT group, the dosimetric parameters of IBCL seeds, such as prostate V200 (*p* = 0.013) and V250 (*p* = 0.010) and urethra D30 (*p* = 0.038), were better than those of free seeds on intraoperative dosimetry. Furthermore, even on post-implant dosimetry, prostate D90 (*p* = 0.004), V150 (*p* = 0.001), and homogeneity index (HI, *p* = 0.001), as well as V200 (*p* = 0.001) and V250 (*p* = 0.020), and urethra D5 (*p* = 0.008) as well as D30 (*p* = 0.003) had a better dosimetric quality in IBCL seeds than in free seeds. There was no significant difference in the operation time between free seeds and IBCL seeds in each PI and PI + EBRT group.

**Conclusions:**

Our results reveal that greater dosimetric benefits could be obtained using IBCL seeds in the case of permanent implantation with a lower prescribed dose, such as PI + EBRT, rather than PI alone.

## Background

Permanent iodine-125 (I-125) seed implantation is a well-established curative treatment option for localized prostate cancer and can deliver a high, localized radiation dose to the tumor with excellent biochemical control of disease [1, 2]. Prostate brachytherapy also has advantages over external beam radiation therapy (EBRT) because of its ability to overcome problems of organ movement. The techniques of seed implantation have been developed in various ways, for example, planning methods (intraoperative real-time planning or inverse planning) and the type of seeds (free or stranded). The difficulty in maintaining the dosimetric quality differs along with the condition of the patient. To confirm the actual dose delivered or identify any deviation from the treatment plan, postoperative dosimetry is recommended [3]. Compared with free seeds, stranded seeds significantly decrease the incidence of seed migration [4–6]. Each type of seed also has identical characteristics in terms of dose quality and distribution. Zaulus et al. first reported that the delivery system with intraoperatively built custom-linked (IBCL) seeds allows the stable implantation of seeds with less seed migration [7]. Ishiyama et al. revealed that dosimetric change due to the selection of seed type with free and IBCL seeds made an impact on the clinical outcome of the study [8].

Recently, combined therapy with lower-prescribed-dose prostate permanent I-125 seed implantation (PI) and EBRT, involving 110-Gy PI followed by 45-Gy EBRT (PI + EBRT), resulting in a total biological effective dose with an α/β value of 2 Gy (BED_2_) = 220–240 Gy, is often performed in patients with high-risk prostate cancer and has shown better biochemical control [1, 9–11]. Along with constant standardization of radioactivity per seed, PI with a higher prescribed dose requires a higher total seed number to achieve an adequate dose quality for the prostate. On the other hand, in PI for high-risk prostate cancer, a comparatively higher peripheral seed number is required to be arranged near the capsule of the prostate because of the risk of tumor relapse from the prostate periphery. As a result, 110-Gy PI with EBRT in high-risk patients might require a greater deal of technical implantation skills than 160-Gy PI in intermediate- and low-risk patients.

Therefore, there is the possibility that the impact of seed type, such as free seeds or stranded seeds, on dose distribution varies according to a dose prescription or a seed number required in each case. However, to the best of our knowledge, the dosimetric advantage conferred by intraoperative-built custom-linked seeds has not been fully elucidated for brachytherapy with a lower prescribed dose similar to that in PI + EBRT cases. In the current analysis, we evaluated the differences in the impact of IBCL seeds on dose quality between two different prescribed doses: 110 Gy in PI + EBRT and 160 Gy in PI on transperineal interstitial prostate brachytherapy.

## Methods

A total of 63 patients with newly diagnosed, localized prostate cancer of clinical stage T1c–T3a with prostate-specific antigen (PSA) levels of 4.0–89.1 ng/mL and a Gleason score (GS) of 6–9 were treated using transperineal interstitial prostate brachytherapy with radioactive I-125 from November 2012 to October 2014. Of these patients, 39 with high-risk prostate cancer were treated with 110-Gy PI, followed by EBRT 1 month after implantation (PI + EBRT), and 24 with low- and intermediate-risk were treated with 160-Gy PI alone. Implantations for the PI + EBRT group with free seeds were conducted as a cohort study, and a portion of the records were extracted with regards to the period during which medical staffs of a specific composition joined PI for this study. For comparison with our conventional technique with free seeds and the first experience of the use of IBCL seeds, these patients’ records were retrospectively reviewed during the specific period in which the learning curve of prostate brachytherapy in our institution had already reached an equilibrium state. Each implantation was conducted by the same composition of medical staff. This study was approved by our institutional review board. All patients were included in this analysis except for one patient in the PI + EBRT group who was excluded because of a partial deficit in clinical data.

All patients also underwent neoadjuvant hormonal therapy. The patient and treatment characteristics are shown in Table 1. One month before implantation, preoperative planning was performed with sagittal transrectal ultrasound images taken in the range of 120°–140° at an angle of 1° each, which were captured by a treatment planning system (SPOTPRO™, Nucletron, Veenendaal, Netherlands). The entire prostate, urethra along with a balloon catheter, and rectal anterior wall were contoured using reconstructed axial images at 2.5-mm intervals. The clinical target volume (CTV) was defined as the prostate with no margin beyond the organ. The number of seeds for treatment with each prescribed dose was then determined. Patients in the low- and intermediate-risk group received a total dose of 160 Gy. Patients in the high-risk group received a total dose of 110 Gy, followed by 45 Gy EBRT 1 month after implantation. Treatment planning with 160 Gy or 110 Gy was performed using a real-time peripheral loading approach without inverse optimization. The other dosimetric targets were used for prostate implants as follows: the minimal dose received by 90% of the CTV (D90) > 100% of the prescribed dose, irradiated dose to 30% of the urethral volume (UD30) ≤ 200 Gy for 160-Gy and 10% of the urethral volume (UD10) ≤ 200 Gy for 110-Gy brachytherapy, and rectal volume receiving the prescribed dose (RV100) < 1 cm^3^ for both prescribed doses. In the period from November 2012 to February 2014, the patients received OncoSeed™ I-125 implants (GE Healthcare, Medi-Physics Inc., Arlington Heights, IL) as a type of free I-125 seeds using the Mick applicator (Mick Radio-Nuclear Instruments, Mount Vermont, NY), and in the next period from February 2014 to October 2014, the patients received Quicklink® I-125 implants as a type of intraoperatively linked I-125 seeds using a Quicklink® device (CR Bard, Covington, GA). All types of seeds were manually delivered, and two specific physicians and two physicists were involved in these implantations in an alternating fashion.

**Table 1:** The patient and treatment characteristics (*n* = 62)

Characteristics	PI group			PI + EBRT group		
	Free seed	IBCL seed	*p* value	Free seed	IBCL seed	*p* value
Patients	14	10		25	13	
Age (years)	70.5 ± 5.9	72.5 ± 6.4	0.859	73 ± 4.1	735 ± 4.5	0.093
Intra-operative prostate volume (cm^3^)	18.9 ± 4.7	18.5 ± 10.6	0.595	18.9 ± 7.7	16.7 ± 9.7	0.734
Post-operative prostate volume (cm^3^)	16.4 ± 5.6	17.8 ± 9.7	0.507	16.4 ± 6.9	16.5 ± 10.0	0.326
Initial PSA (ng/mL)	7.1 ± 3.1	8.9 ± 4.6	0.344	8.8 ± 16.4	13.4 ± 27.9	0.424
Clinical stage			*NA*			0.077
T1c-T2a	12	9		11	5	
T2b	2	1		3	6	
T2c-T3a	0	0		11	2	
Gleason sum			*NA*			*NA*
≤6	1	0		0	0	
7	13	10		2	1	
≥8	0	0		23	12	
Risk group			*NA*			*NA*
Low	1	0		0	0	
Intermediate	13	10		0	0	
High	0	0		25	13	
Implanted seed number	65.5 ± 8.8	63.5 ± 11.2	0.780	51 ± 10.3	47 ± 9.6	0.318
Total activity (MBq)	748 ± 141	715 ± 250	0.652	561 ± 134	517 ± 143	0.465

*Abbreviations*: *PSA* Prostate-specific antigen

All values were expressed as median ± standard deviation

Postoperatively, fluoroscopic images of the pelvis were obtained for confirmation of the number of implanted seeds. All patients underwent a series of radiographs, including chest and kidney–ureter–bladder radiographs, to identify the sites of seed loss and migration. For the post-implant dosimetric evaluation, all patients underwent computed tomography scanning of the pelvis with 1.25-mm-thick slices the next day and 30 days after implantation. The dosimetric parameters with regard to intraoperative images and images at 30 days after implantation were calculated for dosimetric quality and dose distribution as intraoperative values and post-implant values, respectively, such as prostate D90 (Gy, the radiation dose to 90% of the prostate volume), V100 (%, the percentage of the prostate volume receiving 100% of the prescribed dose), V150 (%, the percentage of the prostate volume receiving 150% of the prescribed dose), V200 (%, the percentage of the prostate volume receiving 200% of the prescribed dose), V250 (%, the percentage of the prostate volume receiving 250% of the prescribed dose), urethra D5 (Gy, the radiation dose to 5% of the urethral volume), urethra D30 (Gy, the radiation dose to 30% of the urethral volume), rectum V100 (%, the percentage of the rectal volume receiving 100% of the prescribed dose), and HI (%, the homogeneity index). V150, V200, and V250 were calculated to evaluate the existence of local “hot spots.” HI was calculated for the fraction of the prostate volume receiving between 100% and 150% of the prescribed dose as described by Saw et al. [12]. Then, HI was defined as

$$ HI=\frac{V100-V150}{V100}\times 100\% $$

Seed migration was also scored when seeds were confirmed to have been localized in the chest and abdomen or separated by 1 cm from the seed cluster in the pelvis. Seeds placed into the bladder and seminal vesicles were not scored as seed migration but as seed loss.

### Statistical analysis

All parameters were calculated for both groups. Statistical analysis was performed using the χ^2^ test or Fisher’s exact test for qualitative variables and the Student’s *t*-test or Mann–Whitney U test for quantitative variables, depending on the data distribution. The significance level was set as *p* < 0.05.

## Results

As shown in Table 1, the numbers of patients implanted with free seeds and IBCL seeds were 14 (58.3%) and 10 (41.7%), respectively, in the PI group and 25 (65.8%) and 13 (34.2%), respectively, in the PI + EBRT group. There were no significant differences in patient and treatment characteristics between patients implanted with free seeds and IBCL seeds in each group. However, only for analyses including all seed types in comparisons between the PI and PI + EBRT groups, there were significant differences in the GS (*p* < 0.001), clinical T stage (*p* < 0.001), number of seeds (64 ± 9.7 vs 50 ± 10.1, *p* < 0.001), and total activity (748 ± 189 mCi vs 550 ± 137 mCi, *p* < 0.001) as expected, although the parameter of the D’Amico risk classification was not evaluated because of the extreme data distribution.

**Fig. 1:**
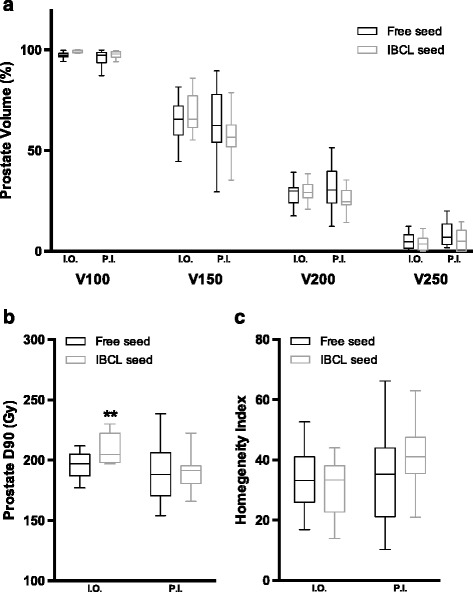
Changes in dosimetric parameters for the PI group with 160-Gy PI. **a** The percentages of the prostate volume receiving each percentage of the prescribed dose. **b** The intraoperative and post-implant D90 prostate values. **c** The homogeneity index for free and IBCL seed groups. The spread of the data is denoted by a box and whiskers plot: box limits represent the 25th and 75th percentiles; the line within the box represents the median; whisker ends represent the 1st and 99th percentiles. *p < 0.05, **p < 0.01, in comparison with free seed and IBCL seed in each I.O. and P.I. Abbreviations: I.O. = intraoperative dosimetry; P.I. = postimplant dosimetry

In the PI group, no impact of differences in seed type was detected on the parameters of CTV dose distribution. However, only for IBCL seeds, prostate V150 and V200 were decreased on post-implant dosimetry 1 month after implantation compared with intraoperative dosimetry and the changes were significant (57.3 ± 11.1% vs 68.3 ± 10.3%, with *p* = 0.006 for V150; 25.7 ± 6.1% vs 29.5 ± 5.7%, with *p* = 0.030 for V200, respectively, Fig. 1a). Although the parameter of prostate D90 in cases with IBCL seeds was significantly higher than that in cases with free seeds on intraoperative dosimetry (209.1 ± 12.6 Gy vs 195.8 ± 10.3 Gy, *p* = 0.009), this difference disappeared on post-implant dosimetry 1 month after implantation (190.1 ± 15.2 Gy vs 190.3 ± 23.6 Gy, *p* = 0.9783, Fig. 1b). The values of HI, which indicate the homogeneity of the CTV dose, did not differ between free and IBCL seeds on either intraoperative dosimetry (33.9 ± 10.0 vs 31.1 ± 10.0, *p* = 0.509) or post-implant dosimetry (33.6 ± 15.5 vs 41.3 ± 10.9, *p* = 0.191, Fig. 1c). For the evaluation of critical organs as shown in Table 2, although a positive effect of the use of free seeds on rectum V100 (*p* = 0.026) was detected, this effect was not seen on post-implant dosimetry (*p* = 0.119). There were no differences in urethra D30 (*p* = 0.398, and *p* = 0.159) and D5 (*p* = 0.236, and *p* = 0.488) between these seed types on intraoperative and post-implant evaluation, respectively.

**Table 2:** The intra-operative and post-implant dosimetric values for critical organs in PI group (*n* = 24)

Risk organs	Intra-operative values			Post-implant values		
	Free seed	IBCL seed	*p* value	Free seed	IBCL seed	*p* value
Urethra						
D30	226.9 ± 23.4	223.7 ± 14.3	0.398	248.9 ± 35.8	219.9 ± 23.6	0.159
D5	245.7 ± 37.3	235.1 ± 15.1	0.236	278.6 ± 44.0	255.6 ± 46.7	0.488
Rectum						
V100	0.07 ± 0.16	0.30 ± 0.32	0.026	0.14 ± 0.13	0.23 ± 0.35	0.119

*Abbreviations*: *PI* Permanent seed implantation, *IBCL* Intraoperatively built custom-linked

All values were expressed as mean ± standard deviation

**Fig. 2:**
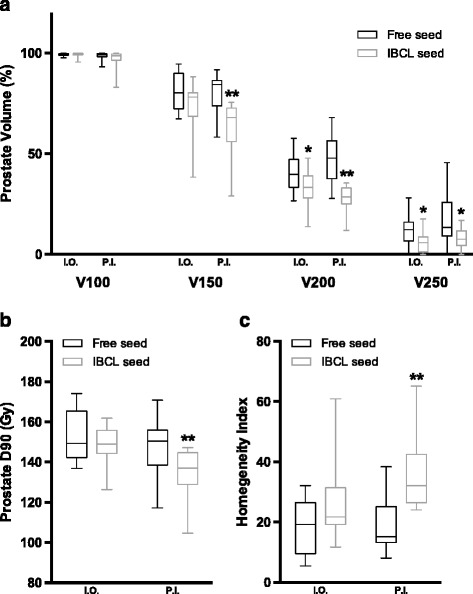
Changes in dosimetric parameters for the PI + EBRT group with 110-Gy PI. **a** The percentages of the prostate volume receiving each percentage of the prescribed dose. **b** The intraoperative and post-implant D90 prostate values. **c** The homogeneity index for free and IBCL seed groups. The spread of the data is denoted by a box and whiskers plot: box limits represent the 25th and 75th percentiles; the line within the box represents the median; whisker ends represent the 1st and 99th percentiles. **p < 0.01, in comparison with free seed and IBCL seed in each I.O. and P.I. Abbreviations: I.O. = intraoperative dosimetry; P.I. = postimplant dosimetry

In the PI + EBRT group, the parameters of prostate V200 and V250 in cases with IBCL seeds were significantly improved compared with cases with free seeds intraoperatively (33.4 ± 8.6% vs 40.8 ± 9.0% with *p* = 0.013 for V200, and 5.7 ± 5.2% vs 12.0 ± 7.2% *p* = 0.010, respectively). These impacts of using IBCL seeds on the improvement of dose distribution became more prominent with fine values of prostate V150 (62.8 ± 12.9% vs 80.2 ± 10.0%, *p* = 0.001), V200 (28.0 ± 6.5% vs 47.5 ± 12.7%, *p* = 0.001), and V250 (8.3 ± 5.4% vs 17.4 ± 12.9%, *p* = 0.020) in post-implant dosimetry on day 30. For IBCL seeds, prostate V150 and V200 were decreased on post-implant dosimetry 1 month after implantation compared with intraoperative dosimetry with significant changes (62.8 ± 12.9% vs 74.0 ± 12.6% with *p* < 0.001 for V150, and 28.0 ± 6.5% vs 33.4 ± 8.6% with *p* < 0.001 for V200, respectively, Fig. 1a). On the contrary, for free seeds, prostate V200 was increased on post-implant dosimetry compared with intraoperative dosimetry (47.5 ± 12.7% vs 40.8 ± 9.0%, *p* < 0.001, Fig. 2a). Although there was no difference in prostate D90 between free seeds and IBCL seeds on intraoperative evaluation (153.0 ± 11.8 Gy vs 149.1 ± 9.1 Gy, *p* = 0.297), the value was decreased preferentially in the IBCL seed group on post-implant evaluation, resulting in a significant difference (148.3 ± 13.7 Gy vs 134.5 ± 12.1 Gy, *p* = 0.004, Fig. 2b). The values of HI did not differ between free seeds and IBCL seeds on intraoperative dosimetry (18.9 ± 8.8 vs 25.4 ± 12.5, *p* = 0.067) but were improved with IBCL seeds on post-implant evaluation (18.7 ± 9.3 vs 35.7 ± 11.7, *p* < 0.001, Fig. 2c). In the assessment of critical organs as shown in Table 3, although a tendency toward an improvement in rectum V100 was seen in cases with IBCL seeds on intraoperative dosimetry (*p* = 0.074), this effect was diminished on post-implant dosimetry (*p* = 0.306). The parameters of urethra D30 were significantly improved in cases with IBCL seeds compared with cases with free seeds. These impacts of using IBCL seeds on the improvement of the urethral dose were greater on post-implant dosimetry (*p* = 0.003 for D30, and *p* = 0.008 for D5).

**Table 3:** The intra-operative and post-implant dosimetric values for critical organs in PI + EBRT group (*n* = 38)

Risk organs	Intra-operative values			Post-implant values		
	Free seed	IBCL seed	*p* value	Free seed	IBCL seed	*p* value
Urethra						
D30	174.5 ± 14.3	166.0 ± 14.2	0.038	195.1 ± 32.7	165.2 ± 23.2	0.003
D5	190.9 ± 19.4	184.4 ± 20.4	0.29	230.1 ± 36.3	200.3 + 32.6	0.008
Rectum						
V100	0.42 ± 0.44	0.13 ± 0.36	0.074	0.29 ± 0.35	0.31 ± 0.20	0.306

*Abbreviations*: *PI* Permanent seed implantation, *EBRT* External beam radiotherapy, *IBCL* Intraoperatively built custom-linked

All values were expressed as mean ± standard deviation

One month after implantation, seed migration was detected in 4 (28.6%) and 0 (0%) patients with free seeds and IBCL seeds, respectively, in the PI group and 6 (24.0%) and 0 (0%) patients, respectively, in the PI + EBRT group.

**Fig. 3:**
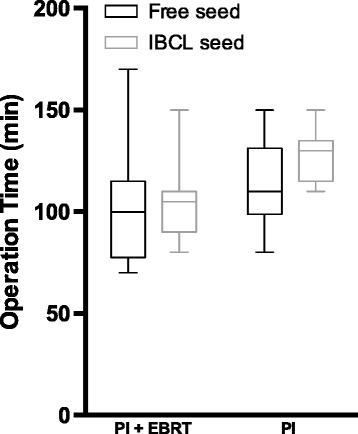
Comparison of operation times (min). The box indicates the 25th and 75th percentiles; the horizontal line in the box represents the median. Whisker ends represent the 1st and 99th percentiles

A record of operation time in one IBCL seed case of the PI group was lost, and the numbers of patients treated with free seeds and IBCL seeds for the analysis of operation time were 14 (60.9%) and 9 (39.1%), respectively, in the PI group and 25 (65.8%) and 13 (34.2%), respectively, in the PI + EBRT group. Differences were not detected between cases with free seeds and IBCL seeds in either the PI (100 ± 28.3 vs 105 ± 17.7, *p* = 0.765) or the PI + EBRT group (110 ± 22.0 vs 130 ± 13.0, *p* = 0.097, Fig. 3).

## Discussion

Intraoperative prostate shape distortion and swelling is generally experienced during permanent brachytherapy of the prostate. These volumetric changes in the prostate are reported by McLaughlin et al. to have an impact on dosimetry of permanent implantation [13]. Although the use of stranded seeds is expected to prevent intraprostatic seed displacement and migration [14, 15], Pinkawa et al. reported that even using stranded seeds generates negligible intraprostatic seed displacement, which correlates with the influence of dosimetry [16]. Chng et al. revealed that intraprostatic seed displacement and unexpected seed misorientation, even using stranded seeds, may also decrease dose quality [17]. Fagundes et al. and Lin et al. reported that stranded seeds improve the dosimetry of prostate V100 and D90 significantly, compared with free seeds [18, 19]. In agreement with these results, Heysek et al. presented that D90 was improved by the use of stranded seeds [20]. On the other hand, Salbishkumar reported that dosimetric advantages were not observed at day 7, although there were advantages of the use of stranded seed in V100 and D90, and a better dose distribution in V150 was also attained with stranded seeds compared with free seeds at day 0 after implantation. [6]. As in these reports, there is no evidence that stranded seeds are necessarily superior to free seeds.

In this study, we are the first to report the dosimetric advantage of intraoperatively real-time custom-linked seeds in PI with a lower prescribed dose. This type of seed was first reported by Zaulus et al. [7]. They confirmed the validity of the use of IBCL seeds with regards to dosimetry in PI prescribed 160 Gy compared with free seeds. Later, Jarusevicius et al. reported in a cohort study with 230 patients that IBCL seeds conferred a dosimetric advantage over free seeds in PI prescribed 160 Gy [21]. However, Ishiyama et al. could not observe the advantage of the use of IBCL seeds in PI prescribed 145 Gy, although IBCL seeds prevented seed migration [8]. In agreement with a previous report, Katayama et al. reported no evidence of improvement in prostate D90, which was observed at day 30 in PI prescribed 144 Gy [22]. Altogether, we can find no evidence in these reports as to whether a dosimetric advantage exists in PI with IBCL seeds. These results suggest that the impact of seed type, such as free or IBCL seeds, on dose quality differs between institutions due to the difference in methodology for placement of the seeds and the shape of the dose distribution. In this study, we adopted a real-time peripheral loading approach without inverse optimization, and achieved the improvement of dosimetry for organs at risk, such as urethra and rectum, as well as the CTV dose homogeneity. I-125 emits low energy photons with average 28 keV, which is lower than the photons of EBRT, resulting in limited tissue penetration of photons [23]. Then, effective radiation doses are confined to a few millimeters beyond the target with less dose to peripheral tissues. Therefore, our results imply that brachytherapy, particularly with stranded sources, potentially give larger dose to peripheral margin more safely than EBRT, with less dose to an adjacent organ one wants to avoid (i.e. rectum and bladder) that is not possible with EBRT because of its higher energy.

As mentioned above, even though the use of stranded seeds potentially generates seed displacement [16], the method of real-time treatment plan refinement, such as seed localization and dosimetric modification, is more suitable for uncertainty in PI. Therefore, IBCL seeds, which are real-time built depending on the situation, are theoretically superior to preoperatively built stranded seeds. In this study, we determined that the use of IBCL seeds conferred an advantage on dosimetry in PI with a lower dose followed by EBRT, which is continuously observed until 1 month after implantation but no advantage in PI as a monotherapy with a higher dose. A possible reason for this is that the deficit in dosimetry caused by seed displacement and misorientation might be compensated for easily, even by the use of free seeds, due to the small contribution of each seed to the total radioactivity in PI with a higher dose, and that compensation of the deficit in dosimetry might be accomplished only by the use of IBCL seeds, which might be able to overcome the great challenge caused by the large contribution of each seed to the total radioactivity in PI with a lower dose.

The number of patients reviewed in this study was limited because the specific period was extracted in which prostate brachytherapy in our institution had already reached an equilibrium state in terms of technical skills, and all procedures were conducted by the same composition of medical staff. However, even though few patients were included, the advantage of IBCL seeds was observed; thus, a larger impact of IBCL is expected with regards to PI with a lower dose.

In the present study, no seed migration was observed in the IBCL group. The incidence of seed migration was widely reported as 0–69.4% in patients treated with 145- or 160-Gy PI [4, 5, 24–31]. Especially in lower-dose PI cases, such as those treated with 110 Gy, there are few reports with regards to seed migration [32]. The utilization of stranded seeds is reported to prevent seed migration [4, 5, 29]. As with stranded seeds, it is reported that IBCL seeds also eliminate the occurrence of seed migration. In agreement with these results, from our results showing no migration in the IBCL group, a reduction of the risk of seed embolization is expected.

## Conclusions

In conclusion, our study shows the dosimetric advantage of IBCL seed implantation compared with free seed implantation, which is more effective in PI prescribed with a lower dose than a higher dose.

## Abbreviations

CTV: Clinical target volume
EBRT: External-beam radiation therapy
GS: Gleason score
HI: Homogeneity index
I-125: Iodine-125
IBCL: Intraoperatively built custom-linked
NA: Not applicable
PI: Permanent implantation
